# The Actual Cautery in Cancer of the Uterus

**Published:** 1882-11

**Authors:** O. Stroinski

**Affiliations:** Chicago


ARTICLE V.

### THE ACTUAL CAUTERY IN CANCER OF THE UTERUS. By O. STROINSKI, M.D., Chicago.

There are two essential points in cancer of the uterus, which have been hitherto entirely ignored by gynæcologists, and which play an important role in the surgical treatment of this disease. (1). In medullary carcinoma, which is by far the most frequent form of cancer of the uterus, the os-internum is the rubicon which will be seldom passed, either by the cancer of the cervix or by that of the body. (2). In cancer of the cervix uteri there is œdematous sw[illegible]lling of the vaginal wall around the cervix so that the os-externum and the vaginal walls are on a level plane. Ruze and Veit, in their excellent monograph on cancer of the uterus, lately published, have given full attention to the first point. They say that medullary carcinoma, while mostly originating from the glands of the uterine membrane, will be found located either in the body or in the cervix, and that total extirpation is an uncalled for operation, the results being very unfavorable, and amputation of the upper segment being preferable in cancer of the body. I do not think total extirpation of the uterus a suitable operation for cancer of the cervix uteri—every surgeon may settle this question with his own conscience—there being operations less difficult, and prolonging the life of the patient without risk. Amputation of the cervix would be the operation *par excellence*, but the above named swelling of the vaginal walls impedes the operation and makes its performance often impossible without injury to the bladder. And there is yet another point to consider. The cancer of the cervix, while mostly not transgressing the os-internum, invades that part of the cervix which is situated above the vaginal portion of the cervix. German physicians practice, therefore, the funnel-shaped excision of the cervix, and English surgeons scrape out the uterus and apply caustics. The funnel-shaped excision and the scraping out the uterus with the sharp spoon removes a great deal of healthy tissue which would be used as a stratum by the advancing disease. A second operation is hereafter impossible on account of the scarcity of tissue. The caustics, as chromic acid, chloride of zinc, sulphate of zinc, bromide and chian turpentine, which latter is entirely ineffectual, attack the walls of the vagina in the same degree as the uterus, while liquidated in contact with the uterine membrane or tissue. The application of these caustics is also very difficult, it being often impossible to bring them in contact with the diseased part. The actual cautery (ferrum candens) has been in common use in Germany and other parts of Europe. The method which I have adopted after several trials with other operations, is as follows: The index finger is introduced into the cervix, and as much as possible of the growth is removed by the finger. I then take a blunt spoon and scrape out all of the diseased tissues until I feel the muscular coat. From the latter I scrape off the superficial layer, and I then remove the spoon. I then apply the hot iron three or four times, each time examining with the well-oiled finger. From this time on I watch the patient carefully. If there is any new growth, or if the patient complains about sensations usual to this disease, I apply the cautery again. The thermo-cautery of Paquelin I do not use on account of the adhering blood and tissue to the instrument, which makes its use ineffectual. I have the advantage by this method to operate as often as I think it necessary, and I have no loss of healthy tissue. In cautery, I have seen new healthy growths in places where there has been decayed structure. I can furthermore apply this method in cases where the os-externum is apparently in healthy condition and the seat of the disease is located near the os-internum. The women are but the first time afraid of the hot iron, but after they have learned the good effects of it they willingly undergo this but little painful operation. It is necessary to have several irons, the first being always covered with blood and tissue. The application can be easily made in the office, and in order to use it on the diseased point it is necessary to examine the cervix carefully each time. The vagina is not in the least affected, even by taking a common double-bladed speculum. I will relate here but one case, which will show the efficacy of this method.

Mrs. N., fifty years of age, had ceased to menstruate five years ago. One year afterward a severe hæmorrhage set in, which recurred at intervals. Three years ago a fetid liquid began to be separated from the genital parts, and the last three months she was constantly in bed. When examined by me, the woman was exceedingly anæmic and cachetic, pulse 135, hardly perceptible, high fever, especially at night, with intense thirst. She could sleep but one hour at nights. The cervix uteri was wide open; the lips of the vaginal portion thin and flabby, above the vaginal portion in the upper part of the cervix there was a friable growth extending to the os-internum, and filling out that part entirely. The growth was removed by the finger, but the spoon was not used on account of the thinness of the walls. The least force would have opened the sac of Douglas; I applied the hot iron twice, but superficially, and after a week I repeated the application. After two months treatment, the fever and pains were gone and the fetid fluid had changed to a thick, inoffensive liquid. Now after four months the woman looks healthy and has no trouble whatever. In this case, which on account of the serious circumstances, was refused by other gynæcologists to be operated on, no other method could have been applied without injury to the patient.

